# Supplementary material for: Micro triboelectric ultrasonic device for acoustic energy transfer and signal communication
Source: Nat Commun. 2020 Aug 18;11:4143. doi: 10.1038/s41467-020-17842-w (PMC7434882; doi:10.1038/s41467-020-17842-w)
Supplement: Supplementary file 1 — Supplementary Information [file 41467_2020_17842_MOESM1_ESM.pdf]

Supplementary Information (SI)

Micro Triboelectric Ultrasonic Device for Acoustic Energy Transfer and Signal Communication

Chen et al.

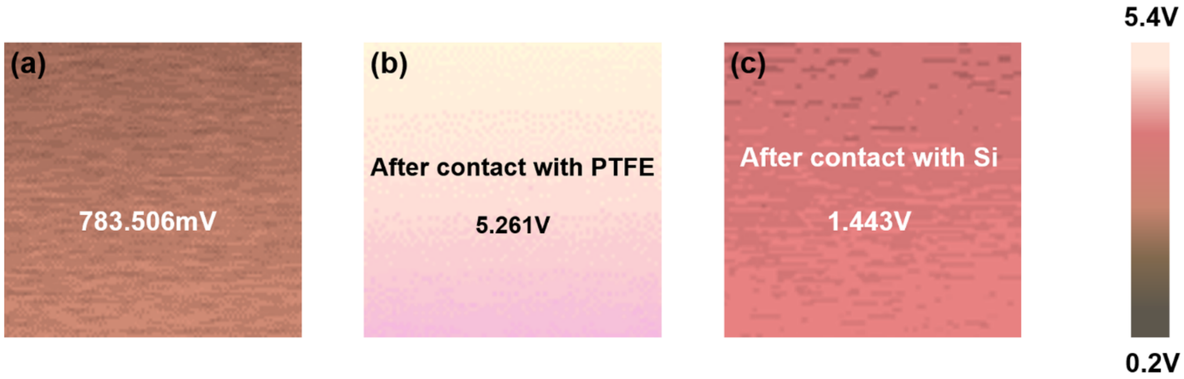

Supplementary Figure 1. SKPM measurements of silicon oxide in different conditions. Surface potential distribution of silicon oxide (a) before contact electrification, (b) after contact with silicon, and (c) after contact with PTFE.

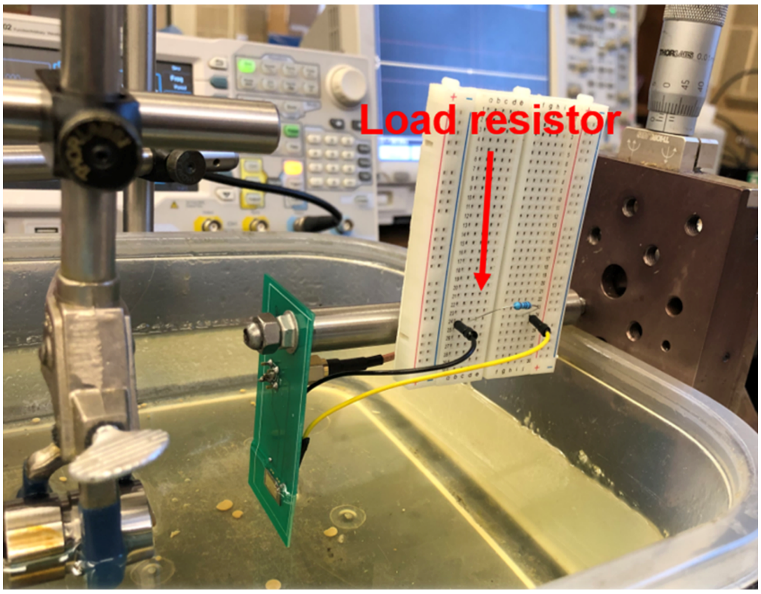

Supplementary Figure 2. Acoustic experimental setup for calculating output power. The μTUD was mounted on a XYZ translation stage with standard micrometers to precisely control the position. Load resistor was connected with the μTUD in series.

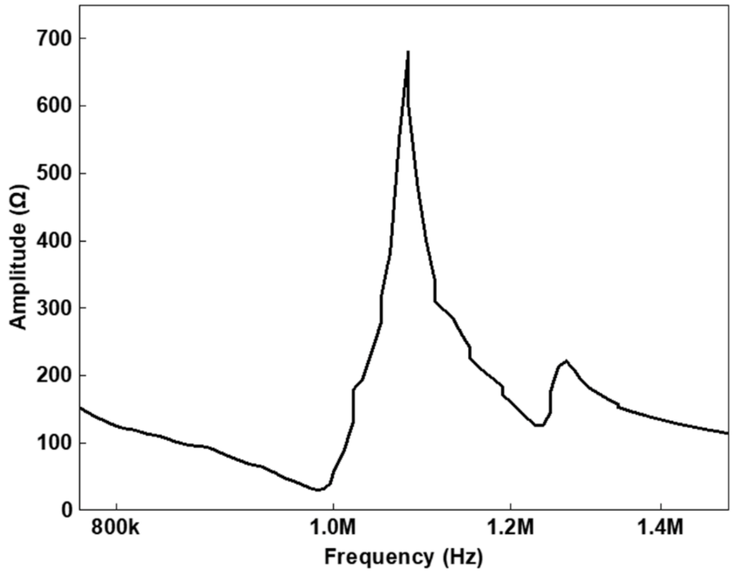

Supplementary Figure 3. Impedance analysis of a commercial ultrasound transducer. Input impedance of the transducer is 57.4 Ω at the frequency of 1 MHz and this impedance is close to the output impedance of the signal generator (50 Ω). Therefore, the impedance should be set to "50 Ω" mode in signal generator.

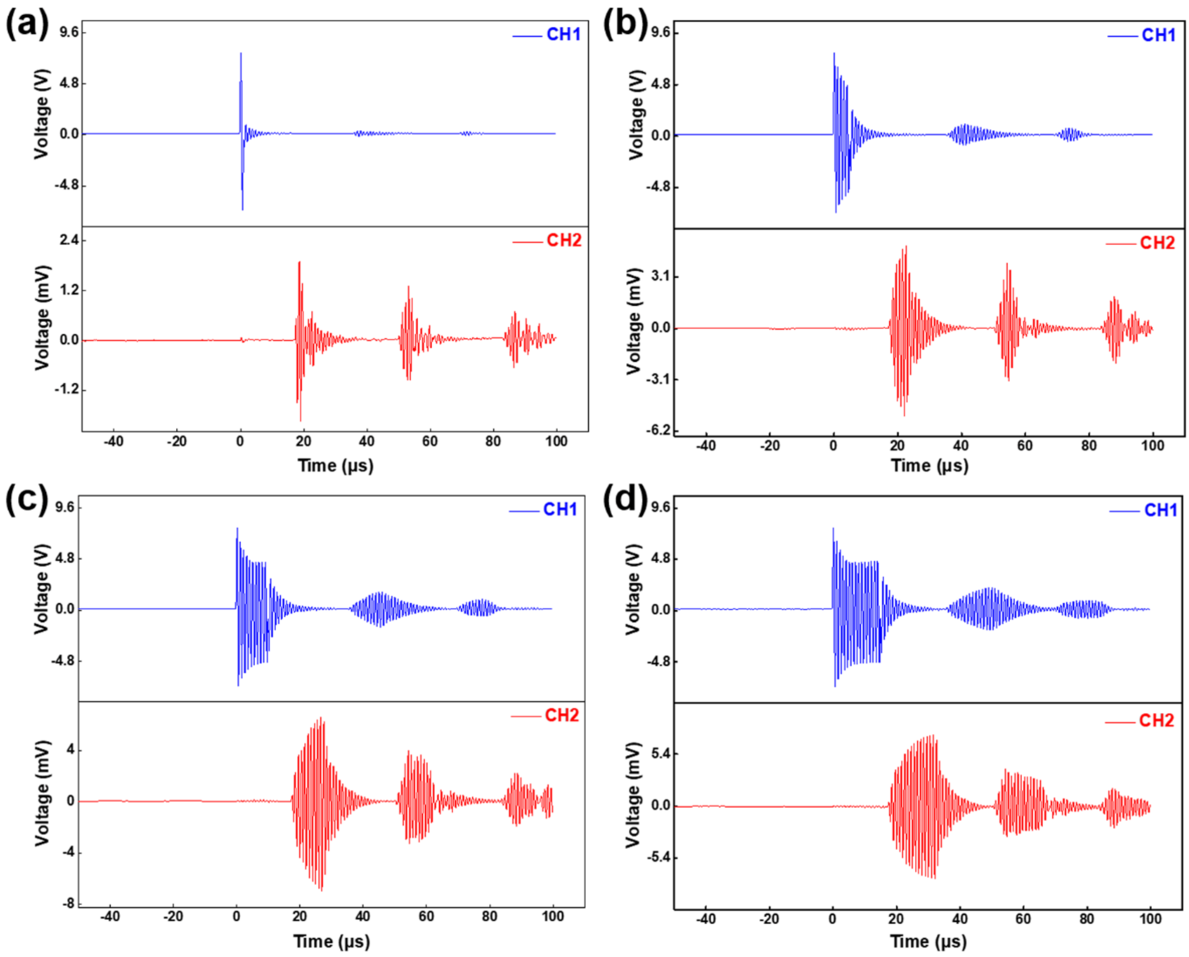

Supplementary Figure 4. CH1 signals and CH2 signals driven by (a) 1-cyc, (b) 5-cyc, (c) 10-cyc and (d) 15-cyc sinusoidal waves (1 MHz, 10 Vp-p, 30 mm separation).

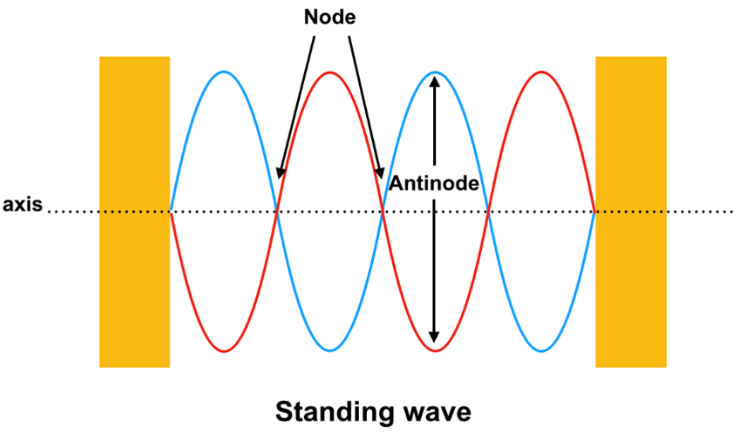

Supplementary Figure 5. Representative schematic of standing wave effect.

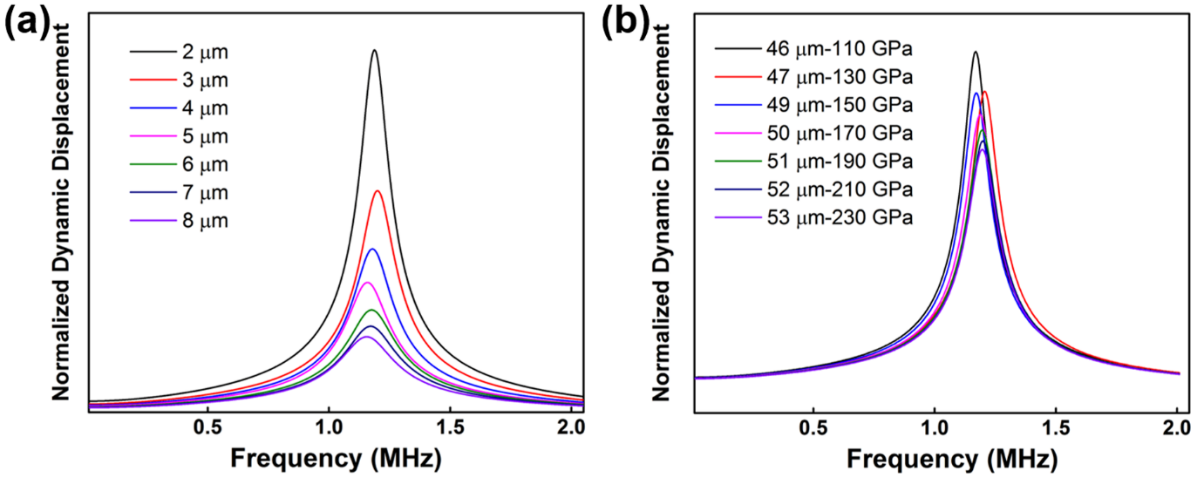

Supplementary Figure 6. Dynamic displacement of membranes. Dynamic displacement of membranes with (a) different thickness and (b) different Young's modulus.

Supplementary Table 1. Acoustic properties of tissues/materials [1,2].

**Tissue/material**	**Sound speed ($m \cdot s^{-1}$)**	**Density ($g \cdot cm^{-3}$)**	**Acoustic impedance (MRayls)**
Water	1480	1	1.48
Vegetable oil	~1500 (measured)	1.070	1.60
Blood	1575	1.055	1.66
Flat	1450	0.95	1.38
Muscle	1575	1.065	1.68
Skin	1730	1.15	1.99

Supplementary Table 2. Parameters for theoretical analysis.

Parameters	Quantity
Thickness of Si layer	2 μm
Thickness of top electrode	150 nm
Young's modulus of Si	170 GPa
Poisson ratio of Si	0.28
Density of Si	2329 $kg\cdot m^{-3}$
Radius of medium	500 μm
Radius of circle	50 μm

Supplementary Table 3. Results of theoretical analysis

		**Static Displacement**	**Resonant Frequency**
(Analytical)		79.4 nm	1.18 MHz
(Numerical)	With top gold layer	74.5 nm	1.17 MHz
	Without top gold layer	79.1 nm	1.19 MHz

Supplementary Table 4. Published data of MEMS-based AET systems for implanted devices.

Reference	Frequency	Transducer type	Medium	Range	Efficiency
Fowler *et al.* [3]	25 kHz	Electrostatic	Air	50 mm	0.42%~1.58%
Horowitz *et al.* [4]	5.2 & 13.6 kHz	PZT thin film	Air	N/A	4×10^{-4}%~0.012%
Shi *et al.* [5]	300 kHz	PZT film	Water	10 mm	0.375%
This work	1 MHz	Triboelectric	Porcine tissue	30 mm	2.1×10^{-4}%

Supplementary Table 5. Parameter combinations of membranes. Different thickness is investigated.

Thickness (μm)	Radius (μm)	Resonant Frequency (MHz)
2	50	1.18
3	63	1.20
4	75	1.19
5	86	1.17
6	95	1.19
7	104	1.19
8	113	1.17

Supplementary Table 6. Parameter combinations of membranes. Different Young's modulus is investigated.

Young's modulus (GPa)	Radius (μm)	Resonant Frequency (MHz)
110	46	1.17
130	47	1.20
150	49	1.17
170	50	1.19
190	51	1.19
210	52	1.20
230	53	1.19

Supplementary Note 1. Analytical model for the static displacement

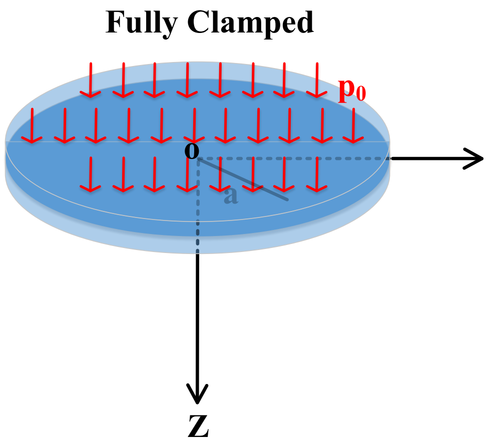

Since the load pressure acting on the circular membrane is symmetrical about the *z* axis, the deflection surface is also symmetrical. Thus, Cylindrical coordinate system is applied and the positive direction of the *z* axis is downward.

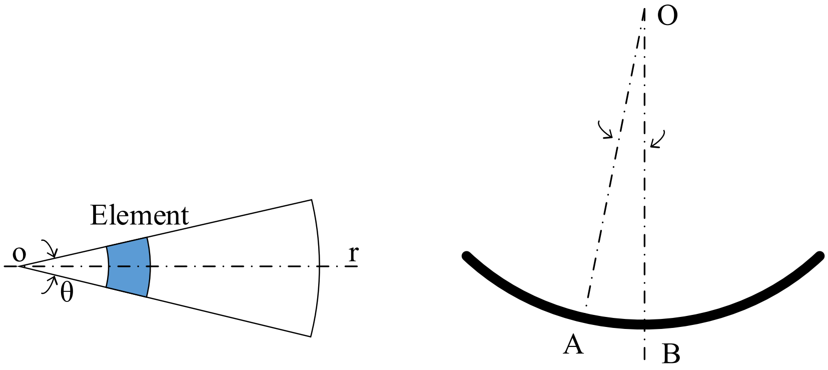

The circular membrane can be divided into an infinite number of small elements and the deflection surface of the membrane is the integration of all the elements. Taking one element of the membrane (blue part in the figure above), the moments along four edges are summed up and the equation of equilibrium is obtained as [6]

$$\left(M_r + \frac{dM_r}{dr}dr\right)(r + dr)d\theta - M_r r d\theta - M_t dr d\theta + Q_r d\theta dr = 0 \quad (1)$$

where M_r is the bending moment per unit length along circumferential sections of the membrane, M_t is the bending moment per unit length along the diametral section *rz* of the membrane, and Q_r the shearing force per unit length of the cylindrical section of radius *r*.

Derived from Kirchhoff-Love plate theory,

$$M_r = -D\left(\frac{d^2w}{dr^2} + \frac{\nu}{r}\frac{dw}{dr}\right) = D\left(\frac{d\varphi}{dr} + \frac{\nu}{r}\varphi\right) \quad (2)$$

$$M_t = -D\left(\frac{1}{r}\frac{dw}{dr} + \nu\frac{d^2w}{dr^2}\right) = D\left(\frac{\varphi}{r} + \nu\frac{d\varphi}{dr}\right) \quad (3)$$

where φ is the small angle between the normal to the small element at point A (corresponding to the blue part) and the axis OB.

By eliminating small quantities of higher order, Equation (1) can be simplified as

$$M_r + \frac{dM_r}{dr}r - M_t + Q_r = 0 \quad (4)$$

Substituting Equation (2) and (3) for M_r and M_t, we obtain

$$\frac{d^3w}{dr^3} + \frac{1}{r}\frac{d^2w}{dr^2} - \frac{1}{r^2}\frac{dw}{dr} = \frac{Q}{D} \quad (5)$$

This Equation (5) can be put in another form

$$\frac{d}{dr}\left[\frac{1}{r}\frac{d}{dr}\left(r\frac{dw}{dr}\right)\right] = \frac{Q}{D} \quad (6)$$

When the membrane is uniformly loaded by the atmospheric pressure (p_0), the shearing force (Q) at a distance (r) from the center of the membrane is defined as

$$2\pi rQ = \pi r^2 p_0 \quad (7)$$

Substituting into Equation (6), we obtain

$$\frac{d}{dr}\left[\frac{1}{r}\frac{d}{dr}\left(r\frac{dw}{dr}\right)\right] = \frac{p_0 r}{2D} \quad (8)$$

The integration of Equation (8) gives

$$w = \frac{p_0 r^4}{64D} + \frac{C_1 r^2}{4} + C_2 \log\frac{r}{a} + C_3 \quad (9)$$

where a is the radius of the membrane. For the circular membrane with clamped edge, the boundary conditions are

$$(w)_{r=a} = 0$$

$$\left(\frac{dw}{dr}\right)_{r=0,a} = 0$$

Applying these boundary conditions to Equation (9), we obtain the value of coefficients

$$C_1 = -\frac{p_0 a^2}{8D}$$

$$C_2 = 0$$

$$C_3 = \frac{p_0 a^4}{D}$$

Substituting coefficients into Equation (9), we find the deflection

$$w = \frac{p_0}{64D}(a^2 - r^2)^2 \tag{10}$$

Supplementary Note 2. Standing wave effect

Standing wave is a stationary wave, where each point on the axis of the wave has constant amplitude. The location, with minimum amplitude, is node while the location, with maximum amplitude, is antinode. The representative schematic is shown as Supplementary Figure 5. Energy fluctuates along the axis: nodes have the maximum acoustic energy and the antinodes have the minimum one. When the receiver is located at the point where the acoustic energy is maximum, it will receive more acoustic energy and convert to more electric energy.

Supplementary Note 3. An estimation of the theoretically maximum efficiency

The electrostatic force, induced by triboelectric charges, will be the main reason to make the membrane collapse. Let's assume an extreme case: After sufficient electrification, the accumulated charges establish an electric field which attracts the membrane to bend. There is a point where electrostatic force is too strong to be balanced by the membrane's restoring force. After this point, the membrane will collapse and the device will fail to work.

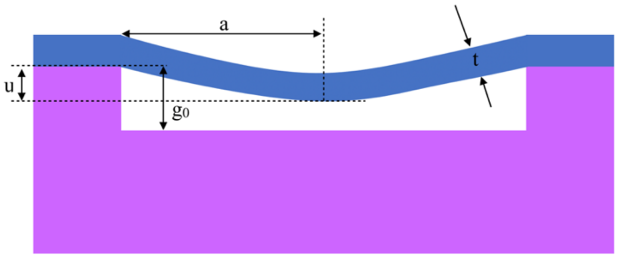

A µTUD cell (gold layer is ignored) is modelled as shown in the figure and mass-spring-damping model is used to analyze its behavior. Here, we assume the total pressure (induced by atmospheric pressure and electric field) on the membrane as *P*. Refer to Supplementary Note 1, the membrane's deflection is defined as

$$u(r) = \frac{Pa^4}{64D}\left(1 - \frac{r^2}{a^2}\right)^2 \tag{11}$$

where *a* is the radius of the membrane and *r* is the distance from the center.

The maximal deflection of the membrane is at *r*=0:

$$u_{max} = \frac{Pa^4}{64D} \tag{12}$$

The average deflection of the membrane is defined as

$$u_{avg} = \frac{\int_0^a 2\pi r u(r)dr}{\pi a^2} = \frac{Pa^4}{192D} = \frac{u_{max}}{3} \tag{13}$$

In the case of small deflection (much smaller than the membrane's thickness), linear system is assumed and the restoring force exerted on the membrane can be written in terms of the average deflection and a linear equivalent spring constant (*k*):

$$F_m = k \cdot u_{avg} \tag{14}$$

Deriving from $P\pi a^2 = k \cdot u_{avg}$, the equivalent spring constant is obtained as

$$k = \frac{\pi a^2 P}{u_{avg}} = \frac{192\pi D}{a^2} \tag{15}$$

To simplify the calculation, we assume the μTUD as a parallel plate capacitor and two contact surfaces are uniformly charged. The critical voltage is defined as [7]

$$V_{PI} = \sqrt{\frac{8}{27}\frac{k\left(g_0 - \frac{\pi a^2 P_0}{k}\right)^3}{\varepsilon_0 \pi a^2}} \tag{16}$$

where ε_0 is the vacuum permittivity, g_0 is the depth of cavity and p_0 is the atmospheric pressure. Substituting the parameters in Supplementary Table 2, we can obtain this critical voltage V_{PI}=5.69 V.

Hence, the maximal effective surface charge density ($\boldsymbol{\sigma}_{max}$) is

$$\sigma_{max} = \frac{V_{PI} \cdot \frac{\varepsilon_0 \pi a^2}{\frac{2}{3} g_0}}{\pi a^2} = \frac{3\varepsilon_0 V_{PI}}{2 g_0} = 840\ \mu C \cdot m^{-2} \tag{17}$$

Therefore, as long as the effective surface charge density is less than 840 $\mu C \cdot m^{-2}$, the μTUD can work properly.

According to the reference [8], the open circuit of the μTUD can be defined as

$$V_{OC} = \frac{d\sigma \cdot 2g_0}{\varepsilon_0 (d + 2\varepsilon_r g_0)} \tag{18}$$

where d is the thickness of the silicon oxide layer and ε_r is the relative permittivity of the silicon oxide layer. To simplify the model, we assume the vibration of the membrane is symmetric, so that the maximal separation is $2g_0$. The equation can be rewritten as

$$\sigma = \frac{V_{OC} \varepsilon_0 (d + 2\varepsilon_r g_0)}{2 g_0 d} \tag{19}$$

Based on our experimental results, the open-circuit voltage of the μTUD is 16.8 mV. The actual effective surface charge density is calculated as 2.71 $\mu C \cdot m^{-2}$. There are two reasons for this low surface charge density currently. Firstly, the selection of silicon and silicon oxide sacrifices

some performance of the μTUD. Secondly, the actual contact area is small compared to the membrane size. Thus, triboelectric charges are not sufficiently transferred between two materials.

Based on Equation (18),

$$V_{OC} \propto \sigma$$

So that output power (P) has the relation:

$$P \propto \sigma^2$$

Hence, the energy harvesting efficiency of the μTUD can be theoretically increased by more than 10000 times, reaching ~33%.

Although it is extremely difficult to practically achieve this high energy conversion efficiency, it is still reasonable to achieve a 1~2 V output voltage by proper optimization method, such as adding a charge trapping structure or additional triboelectric layer [9].

Supplementary Note 4. Mechanical parameters of the μTUD

Both material and structure factors may affect the performance of the μTUD. A brief discussion will be given here to guide the design of the μTUD in the future.

Mechanically, the μTUD may fail to work due to the collapse of membrane (stick to the substrate). Because of the wafer bonding technique, there is no wet etching step for cavity formation so that the capillary adhesion is avoided. Moreover, the silicon membrane is the device layer (single crystalline silicon) of SOI wafer and has a very low intrinsic stress. The stress factor can also be ignored in this discussion. Therefore, the main factor contributing to the failure of the μTUD is electrostatic force, induced by the triboelectric charges. A more detailed discussion about the electrostatic force has been provided in Supplementary Note 3.

Refer to the definition of performance figure-of-merit, the material's triboelectric properties play the most important role in determining TENG's output [10]. The improvement of surface charge density will dramatically increase TENG's output power. With same triboelectric materials, increasing the contact area can effectively improve the device's efficiency because more triboelectric charges are transferred between two materials. Numerical analysis (COMSOL) was carried out to investigate the effect of membrane's thickness and Young's modulus, respectively.

To investigate effect of the membrane's thickness, the Young's modulus was set to 170 GPa. Several combinations of membrane parameters were selected to keep the consistent resonant frequency (as shown in the Supplementary Table 5). With same incident acoustic pressure, the displacement responses of the membranes were numerically simulated and the results are shown in Supplementary Figure 6a. It is seen that a thinner membrane gives a larger dynamic displacement in the same conditions. For a given separation between the membrane and the substrate, the larger dynamic displacement can result in a larger contact area.

To investigate the effect of membrane's Young's modulus, the thickness of the membrane was set to 2 μm. Another group of parameter combination were selected, which is shown in Supplementary Table 6. With same incident acoustic pressure, the displacement responses of the membranes were numerically simulated and the results are shown in Supplementary Figure 6b. It is observed that membrane with the lower Young's modulus has a larger dynamic displacement so that a larger contact area can be achieved.

In conclusion, a thinner and softer membrane can contribute to a larger contact area, resulting in a higher efficiency.

Supplementary References

1 Zeqiri, B. Reference liquid for ultrasonic attenuation. *Ultrasonics* **27**, 314-315 (1989).

2 Duck, F. A. *Physical properties of tissues: a comprehensive reference book.* (Academic press, 2013).

3 Fowler, A. G., Moheimani, S. & Behrens, S. An omnidirectional MEMS ultrasonic energy harvester for implanted devices. *Journal of Microelectromechanical Systems* **23**, 1454-1462 (2014).

4 Horowitz, S. B., Sheplak, M., Cattafesta III, L. N. & Nishida, T. A MEMS acoustic energy harvester. *Journal of Micromechanics and Microengineering* **16**, S174 (2006).

5 Shi, Q., Wang, T. & Lee, C. MEMS Based Broadband Piezoelectric Ultrasonic Energy Harvester (PUEH) for Enabling Self-Powered Implantable Biomedical Devices. *Sci Rep* **6**, 24946, doi:10.1038/srep24946 (2016).

6 Timoshenko, S. P. & Woinowsky-Krieger, S. *Theory of plates and shells.* (McGraw-hill, 1959).

7 Nemirovsky, Y. & Bochobza-Degani, O. A methodology and model for the pull-in parameters of electrostatic actuators. *Journal of microelectromechanical systems* **10**, 601-615 (2001).

8 Wang, S. *et al.* Maximum surface charge density for triboelectric nanogenerators achieved by ionized-air injection: methodology and theoretical understanding. *Advanced Materials* **26**, 6720-6728 (2014).

9 Rekhi, A. S., Khuri-Yakub, B. T. & Arbabian, A. Wireless power transfer to millimeter-sized nodes using airborne ultrasound. *IEEE transactions on ultrasonics, ferroelectrics, and frequency control* **64**, 1526-1541 (2017).

10 Zi, Y. *et al.* Standards and figure-of-merits for quantifying the performance of triboelectric nanogenerators. *Nature communications* **6**, 1-8 (2015).
